# Retrieval of Surface Air Specific Humidity Over the Ocean Using AMSR-E Measurements

**DOI:** 10.3390/s8128016

**Published:** 2008-12-08

**Authors:** Masahisa Kubota, Hihara Tsutomu

**Affiliations:** School of Marine Science and Technology, Tokai University/ Orido, 3-20-1, Shimizu, Shizuoka, Shizuoka, JAPAN 424-8610; E-Mail: tsutomu@mercury.oi.u-tokai.ac.jp (T. H.)

**Keywords:** AMSR-E, surface air specific humidity, ocean, microwave radiometer

## Abstract

We have developed a new algorithm to estimate the surface air specific humidity over the ocean from AMSR-E data. It should be noted that remarkably reduced random errors of the estimated surface air specific humidity result from using the surface air specific humidity provided by reanalysis data. We validated our new algorithm using independent ship and buoy data. The bias, RMS error, and correlation coefficient of the products obtained using our algorithm for global buoys are 0.38 g/kg, 0.61 g/kg and 0.99, respectively. It should be noted that surface specific humidity having similar accuracy to the reanalysis data near in situ data could be derived from AMSR-E data by the present algorithm.

## 1. Introduction

Latent heat flux (LHF) is considered to be the most important constituent of ocean surface heat flux, which comprises shortwave radiation, longwave radiation, latent heat flux, and sensible heat flux. This is because LHF contributes to the regulation of global climate by transporting a large amount of heat energy in the form of water vapor from low latitudes to mid and high latitudes. In addition, LHF plays an important role in the global hydrological cycle by transporting water not only from the ocean to the atmosphere but also from one location to another. Therefore, it is necessary to monitor the LHF for better understanding of the variability of global climate.

Recently, the global LHF is estimated by using data from several satellites and the following bulk formula:

$$\text{LHF}=\rho_{a}L_{v}C_{E}U(Q_{s}-Q_{a}),$$

where *ρ_a_* is the density of air, *L_v_* is the latent heat of vaporization, *U* is the near-surface wind speed relative to the surface current speed, *Qs* and *Q_a_* are the surface and saturated specific humidity, respectively, and *C_E_* is the turbulent exchange coefficient for moisture. In order to estimate the amount of LHF, we need to use three kinds of meteorological data such as wind speed, saturated specific humidity, and specific humidity. The saturated specific humidity data can be estimated from the sea surface temperature (SST) data. Several studies have been performed on the development of algorithms for the estimation of specific humidity, e.g.; [1-3]. Most of these algorithms were developed for application to the data of the Defense Meteorological Satellite Program (DMSP)/ Special Sensor Microwave/Imager (SSMI). DMSP/SSMI data are used in many products, e.g.; Japanese Ocean Flux Data Sets with Use of Remote Sensing Observations (J-OFURO) [4], Goddard Satellite-based Surface Turbulent Fluxes (GSSTF2) [5], and Hamburg Ocean Atmosphere Parameters and Fluxes from Satellite Data (HOAPS) [6]. On the other hand, it should be noted that global microwave data can also be obtained using the Advanced Microwave Scanning Radiometer for EOS (AMSR-E). If we can estimate the specific humidity using AMSR-E data, we can reduce the sampling error in the daily mean value of specific humidity because the observation time of AMSR-E (1 a.m. and 1 p.m.) is quite different from those of DMSP/SSMIs (around 6 a.m. and 6 p.m.). However, to date, only [7] has developed an algorithm for estimation of specific humidity using AMSR-E data. They developed the algorithm by assuming that the values of the reanalysis data are the true values. Several previous studies e.g.; [8] and [9] investigated the accuracy of specific humidity data in reanalysis data. However, it is not clear whether reanalysis data are more accurate than satellite-derived data. Therefore, it is necessary to evaluate the accuracy of the air specific humidity derived from AMSR-E data using [7]'s algorithm, by comparing with ocean observation data.

The objective of our study is to develop an algorithm for the estimation of specific humidity using AMSR-E data. We compare our products with other products and validate all these products using buoy data. The data that we have used in this study are described in the next section. Section 3 presents a new algorithm that is developed by using ship observation data, and shows the evaluation of the results derived from the present algorithms using independent ship observation data. Many products, including our products, are evaluated using buoy data in Section 4. Finally the discussion and summary are given in Section 5 and 6, respectively.

## 2. Data

The data obtained by AMSR-E L1B comprise fundamental physical values observed by the instrument, and in the case of AMSR/AMSR-E, they correspond to brightness temperatures. Although the data are not projected onto a map, but stored in the swath form, data location and quality information are also included in AMSR-E L1B data. In this study, we have used the specific humidity data in the reanalysis data of the National Centers for Environmental Prediction (NCEP)/National Center for Atmospheric Research (NCAR) [10] to obtain information about the surface air humidity. We have also used the in situ surface air specific humidity data, which is observed by ships and buoys, of the International Comprehensive Ocean-Atmosphere Data Set (ICOADS) for the construction of the new algorithm. The ICOADS consists of global marine data observed between 1784 and 2007, primarily from ships of opportunity; the data have been collected, edited, and summarized statistically for each month of each year of the period. ICOADS sampling varies regionally ranging from zero to several thousand samples per 2°×2° box per calendar month. Moreover, in many areas there are time-dependent changes in the sampling density [11]. After carrying out QC, the surface specific humidity data of ICODAS are converted to their corresponding values at a height of 10 m using COARE 3.0 [12].

**Figure 1.**
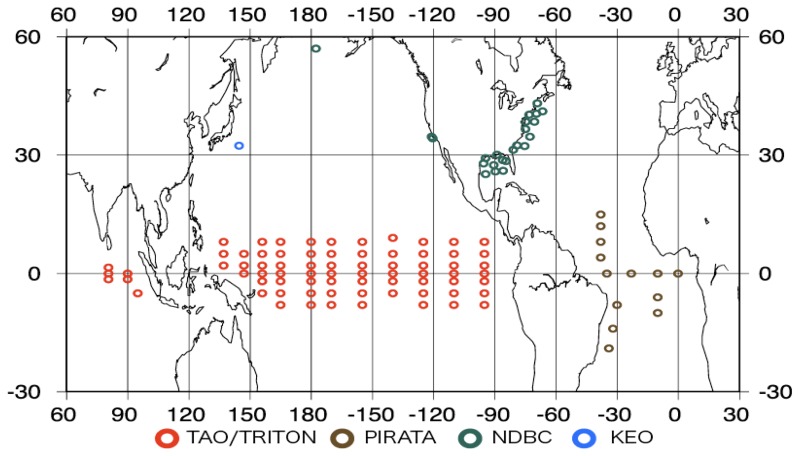
Buoy locations.

We evaluated many products, including the product obtained by our new algorithms, using buoy data. Figure 1 shows the buoy locations. We used the buoy data of the Kuroshio Extension Observatory (KEO) [13], the National Data Buoy Center (NDBC) [14], the Tropical Atmosphere Ocean/Triangle Trans-Ocean Buoy Network (TAO/TRITON), and the Pilot Research Moored Array in the Tropical Atlantic (PIRATA) [15]. We used the data for the year 2004 for analysis, except in the case of the KEO buoy because the KEO has only been carrying out observations since June 2004.

## 3. Development of the new algorithm

We first placed the AMSR-E data and ship observation data in time and space. The temporal and spatial differences between the satellite data and ship observations were limited to less than 30 min and 25 km, respectively. We developed the following regression formula (001) for the collocated satellite and ship observation data. After investigating the most efficient combination of the brightness temperature data observed by the AMSR-E, we finally decided to use all the brightness temperature data in order to obtain an accurate estimation of the surface air specific humidity.

(1) $$Q_{a}^{1}=a_{0}^{1}+a_{1}^{1}T_{6V}+a_{2}^{1}T_{6H}+a_{3}^{1}T_{10V}+a_{4}^{1}T_{10H}+a_{5}^{1}T_{18V}+a_{6}^{1}T_{18H}+a_{7}^{1}T_{22V}+a_{8}^{1}T_{22H}+a_{9}^{1}T_{36V}+a_{10}^{1}T_{36V}+a_{11}^{1}T_{89V}+a_{12}^{1}T_{89H}$$

It should be noted that the vertical profile of humidity affects the accuracy of the estimation of the surface air specific humidity considerably. Since most moisture generally exists near the ground surface, the amount of precipitable water is highly correlated to the surface air specific humidity. Liu [16] performed pioneering work in this field and developed the first algorithm to estimate surface air specific humidity using microwave radiometer data. It should be noted that the difference between in situ and satellite-derived air specific humidity increases if the humidity profile changes because the relation between precipitable water and surface air specific humidity is fixed in this kind of relation. Therefore, we may obtain more accurate specific humidity if we directly use brightness temperature data as the regression formula 001. Reanalysis data may also have different information from that provided by satellite data. Thus, we constructed a second linear regression formula (002) using both the AMSR-E data and Qa (NRA1):

(2) $$Q_{a}^{2}=a_{0}^{2}+a_{1}^{2}T_{6V}+a_{2}^{2}T_{6H}+a_{3}^{2}T_{10V}+a_{4}^{2}T_{10H}+a_{5}^{2}T_{18V}+a_{6}^{2}T_{18H}+a_{7}^{2}T_{22V}+a_{8}^{2}T_{22H}+a_{9}^{2}T_{36V}+a_{10}^{2}T_{36H}+a_{11}^{2}T_{89V}+a_{12}^{2}T_{89H}+a_{13}^{2}Q_{a}(\text{NRA}1)$$

where Qa (NRA1) is surface specific humidity provided by the National Centers for Environmental Prediction (NCEP)/National Center for Atmospheric Research reanalysis (NRA1) data. The regression coefficients are shown in Table 1. The value of a^2^_13_ Qa (NRA1) is not so large compared with other terms because the value of brightness temperatures is one digit larger than that of Qa(NRA1). However, the effect cannot be neglected. The analysis of variance table is given in Table 2 [17]. The statistics prove that 002 is superior.

**Figure 2.**
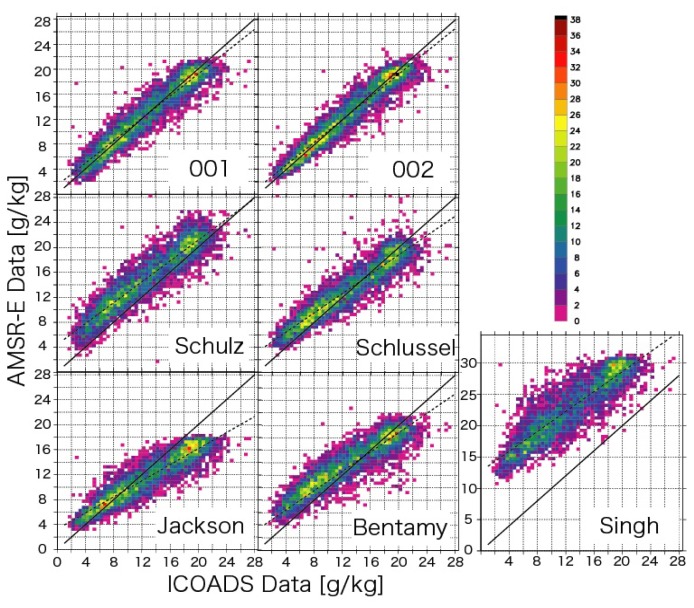
Scatter plots of the surface air specific humidity of the ICOADS data and the data derived from AMSR-E. The color indicates the data number included in a square of 0.5 g/kg × 0.5 g/kg.

We validated the products obtained by the two different formulae using the ICOADS data for 2003–2004. These data are independent of the data used in the construction of the formulae because we developed the formulae using the ICOADS data for 2005. The validation was performed for instantaneous data values. Figure 2 shows the scatter plots of the ICOADS observation data for 2003–2004 and the results of the application of the algorithm to AMSR-E data for 2003–2004. For the purpose of comparison, the results for products obtained by applying the algorithms of [1-2,18-20] to AMSR-E data are also given in the same figure. The statistics are provided in Table 3.

It can be observed that the root mean square (RMS) error remarkably decreases when the algorithm 002 is used. As expected, we can obtain much better results using the new algorithm than the other algorithms. This is expected because the other algorithms were developed not for AMSR-E data but for DMSP/SSMI data. Thus, it can be concluded that we need to develop a different algorithm for each sensor, even if each sensor has channels of similar frequency.

## 4. Comparison with global buoy data

We evaluated various daily products including the present products using buoy data. The statistics are given in Table 4. J-OFURO 2 [21] and HOAPS3 are satellite products, which utilize the data of DMSP/SSMI. We estimated the specific humidity by applying the algorithms of [7] and [18] to AMSR-E data. NRA1, NCEP/Department of Energy reanalysis (NRA2) [22], and Japanese Re-Analysis 25 years (JRA25) [23] data are all types of reanalysis data. Since we compared daily data, the buoy data are averaged into daily mean values and the data in each product are spatially interpolated into buoy location.

It can be observed that the biases of the satellite products are smaller than those of the reanalysis products and our products, while the RMS errors and correlation coefficients of the satellite products are larger and lower than those of the reanalysis products and our products, respectively. It is interesting to note that although our products are satellite products, their statistics are similar to those of reanalysis data. Although buoy data are not assimilated into the present products, the RMS errors are significantly small and the correlation coefficients are considerably high for our products. It can be concluded that we can reduce the RMS errors by inclusion of information about the surface air specific humidity in NRA1.

## 5. Discussions

**Figure 3.**
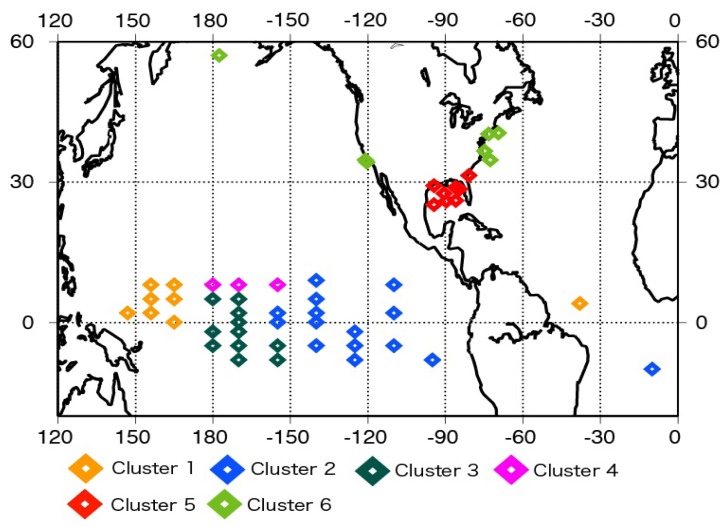
Spatial distribution of buoys in each cluster.

Figure 1 shows that the buoy distribution is inhomogeneous, for example, many buoys exist in the tropical Pacific regions and to the east of the U.S.A. The statistics given in Table 4 are likely to be affected by this imbalance in the buoy number. We carried out cluster analysis for the daily buoy data for 2004. The results are shown in Figure 3.

The clusters are separated on the basis of latitudes. However, it is interesting to note that the clusters in the tropics are separated by longitudes. The characteristics of each cluster are not discussed here and will be discussed in a separate study in the near future. After the cluster analysis, we analyzed the values averaged in each cluster in order to remove the imbalance due to the data number. The results are given in Table 5.

The biases in Table 5 are quite similar to those in Table 4, except for that of JRA25. On the other hand, the RMS errors and correlation coefficients in Table 5 show a remarkable decrease and increase in value, in comparison with the products in Table 4, respectively. This means that it is difficult for all data sets to follow the true variation of air specific humidity in the tropical regions. And we understand the accuracy is considerably good except tropical regions.

**Figure 4.**
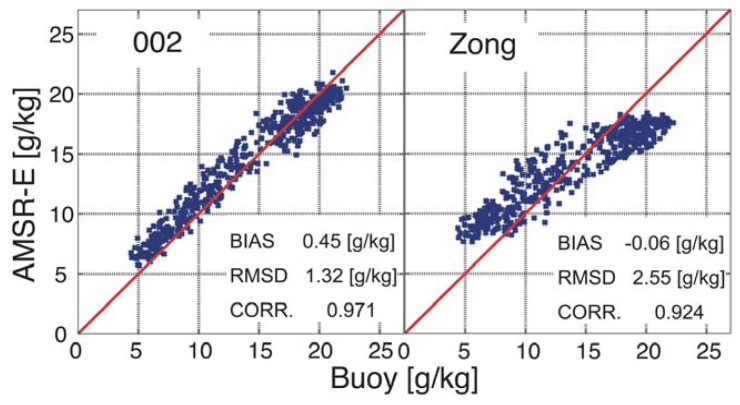
Scatter plots of surface specific humidity derived from KEO buoy and AMSR-E data.

The algorithm developed by [7], which was developed for AMSR-E data, results in a relatively small bias, shown in Table 5, as compared to the bias for the products obtained by our algorithm. Figure 4 shows a scatter diagram for the KEO data and surface air specific humidity obtained by Zong's algorithm and the present algorithm 002.

**Figure 5.**
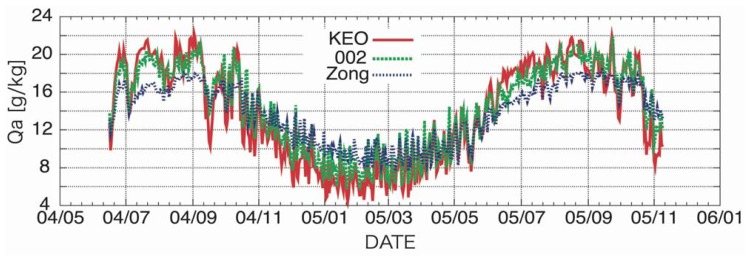
Time variation of surface specific humidity observed by KEO buoy and derived from AME-E data.

The corresponding statistics are also given in the same figure. The RMS errors were 2.55 g/kg and 1.32 g/kg for Zong's algorithm and the regression formula 002, respectively. From this figure, it can be observed that although the bias for Zong's algorithm is considerably smaller than that for 002, the overestimation of small values of specific humidity offsets the underestimation of large values of specific humidity by Zong's algorithm,

Figure 5 shows the time variation of surface air specific humidity. It is clear that Zong's algorithm underestimates the surface air specific humidity in summer and overestimates the surface air specific humidity in winter. The same tendency is found even for 002, but the error is extremely small as compared to Zong' s algorithm. It is concluded that the small bias obtained by using Zong's algorithm is a results of the counterbalance of the underestimation of the specific humidity in summer and overestimation in winter. Thus, it is concluded that a better algorithm could be constructed for the estimation of global surface air specific humidity using AMSR-E data in the present study.

## 6. Summary

We developed a new algorithm to determine the surface air specific humidity from the AMSR-E brightness temperature data by assuming the in situ data of ICOADS for the year 2005 to be true values. The use of surface specific humidity data in NRA1 can significantly reduce random errors. We validated the products obtained by the regression formulae by using two kinds of in situ data—ship data and buoy data. The instantaneous surface air specific humidity data obtained by the new algorithm for the period between 2003 and 2004 are compared with in situ data for the same period. We found that the results yielded much better statistical values than those obtained by applying previous algorithms. It should be noted that most previous algorithms were developed not for AMSR-E but for DMSP/SSMI data. The daily specific humidity data was also validated by comparison with the buoy data. It can be observed that the bias is not as small as that for the satellite and reanalysis products. However, the RMS error is considerably small, i.e.; 0.61 g/kg, as compared to other products. Moreover, the correlation coefficient is considerably high, i.e.; 0.99. Both values are much better than those obtained by the algorithm of [7], which was developed for AMSR-E data. The biases of the products obtained by our algorithms are larger than those of the products obtained by Zong's algorithm for the KEO buoy. However, by observing the time variation of surface air specific humidity observed in Zong' product, we found that the small bias in Zong's product is not homogenous and is caused by an overestimation of the small values of specific humidity in summer and underestimation of the large values of specific humidity in winter Therefore, it can be concluded that the present new algorithm is currently the most superior algorithm to estimate accurate surface air specific humidity using AMSR-E data. Finally the accuracy by the present algorithm is not so different from that by reanalysis data. However, the accuracy shown by the reanalysis data may be much worse in the regions without in situ data because most of in situ data used in the present validation are assimilated into the reanalysis data. Therefore, it is important that surface specific humidity having similar accuracy to the reanalysis data near in situ observation points is obtained by the present algorithm constructed by synthesizing satellite and reanalysis data, because the accuracy of satellite data is considerably homogeneous compared with reanalysis data.

## Figures and Tables

**Table 1.** Regression coefficients.

	**a_0_**	**a_1_**	**a_2_**	**a_3_**	**a_4_**	**a_5_**	**a_6_**
**001**	-92.775	0.092	-0.067	0.199	-0.181	-0.259	0.310
**002**	-49.324	-0.003	0.001	0.136	-0.104	-0.118	0.127
	**a_7_**	**a_8_**	**a_9_**	**a_10_**	**a_11_**	**a_12_**	**a_13_**
**001**	1.451	-0.680	-0.908	0.316	0.173	-0.068	
**002**	0.812	-0.381	-0.524	0.202	0.099	-0.047	0.555

**Table 2.** Analysis of variance (ANOVA) table for simple linear regression of formulae 001 and 002. The column headings df, SS, and MS stand for degree of freedom, sum of square, and mean square, respectively [17].

**001**	**df**	**SS**	**MS**	**F (=MSR/MSE)**

**Total**	5,626	170,096		
**Regression**	12	155,117	12,926 (MSR)	4,845
**Residual**	5,614	14,979	2.67(MSE)	

**002**	**Df**	**SS**	**MS**	**F**

**Total**	5,626	170,096		
**Regression**	13	158,229	12,171 (MSR)	5,946
**Residual**	5,613	11,450	2.05(MSE)	

**Table 3.** Statistics for instantaneous ICOADS specific humidity data and data derived using many different algorithms.

	**001**	**002**	**Schulz**	
**BIAS [g/kg]**	-0.01	-0.03	2.32	
**RMSE [g/kg]**	1.75	1.57	2.17	
**CORRELATION**	0.946	0.957	0.916	
	**Schlussel**	**Bentamy**	**Singh**	**Jackson**

**BIAS [g/kg]**	0.45	0.89	10.12	-1.25
**RMSE [g/kg]**	2.17	2.46	2.65	2.48
**CORRELATION**	0.919	0.903	0.872	0.912

**Table 4.** Statistics for each daily product in comparison to buoys.

	**001**	**002**	**J-OFURO2**	**HOAPS3**	
**BIAS [g/kg]**	0.46	0.38	0.19	-0.21	
**RMSE [g/kg]**	1.23	1.01	1.68	1.57	
**CORRELATION**	0.954	0.970	0.920	0.925	
	**Zong**	**Liu**	**NRA1**	**NRA2**	**JRA25**

**BIAS [g/kg]**	-0.41	0.03	0.72	0.39	0.67
**RMSE [g/kg]**	1.65	1.88	1.13	1.16	0.95
**CORRELATION**	0.923	0.906	0.962	0.959	0.973

**Table 5.** Statistics for daily products that are averaged in each cluster in comparison to buoy data.

	**001**	**002**	**J-OFURO2**	**HOAPS3**	
**BIAS [g/kg]**	0.48	0.38	0.20	-0.20	
**RMSE [g/kg]**	0.80	0.61	1.17	1.15	
**CORRELATION**	0.985	0.992	0.967	0.971	
	**Zong**	**Liu**	**NRA1**	**NRA2**	**JRA25**

**BIAS [g/kg]**	-0.28	0.04	0.71	0.43	0.83
**RMSE [g/kg]**	1.31	1.18	0.72	0.77	0.58
**CORRELATION**	0.967	0.967	0.987	0.985	0.993
